# Institutions – Economic Growth Nexus in Sub-Saharan Africa

**DOI:** 10.1016/j.heliyon.2022.e12251

**Published:** 2022-12-15

**Authors:** Milkessa Temesgen Chomen

**Affiliations:** Department of Agricultural Economics, Ambo University, Ethiopia

**Keywords:** Institution, Economic growth, SSA, System GMM

## Abstract

This research explores the causal link between institutions and economic growth in Sub-Saharan Africa countries. System generalized method of moments (GMM) is applied to fit dynamic models for a panel of 43 countries in the region over thirteen-year period. Major findings include (1) per capita gross domestic product (GDP) is persistent in the region; (2) no significant cause-effect link between institution and economic growth of the countries; and (3) impact of institutions on economic growth appeared affected by control variables. The results show that institutions did not lead to economic growth in SSA countries. The finding adds to the already developing puzzle regarding whether institutions matter for economic development of nations. The study suggests further work in institution-growth studies for proper recognition of institutions role in explaining economic growth differences of countries. In this regard, current research identifies focus areas for future work, which includes considering institutions quality threshold and inclusion of indicators from informal institutions in addition to the formal ones.

## 1 Introduction

The topic of economic growth differences of nations has been the focus of several scholars for quite some time. The quest appears intensified by the extent of differences in the per capita income. Citizens in the richest countries, for instance, earn average income ten times of those in the poorest ones (Mankiw et al., 1995). The growth studies aimed at identifying reasons for such differences. The key question of such works involved what factors determine growth and disparity in economic performance of countries. In attempt to provide answer to this question, growth models included variables from measures of institutions. This is because availability, nature and functioning of institutions determine the organization of production through their effect on investments in human and physical capital. Hence, development is not the mere accumulation of capital, but a process of organizational change (Sarwar et al., 2013). An empirical support to this is Easterly and Levines's (2001) work which concludes “something else” besides Solow's variables explain the growth disparity of nations. Therefore, several studies (e.g. Vijayaraghavan and Ward, 2001; Angeles, 2010; Kilishi et al., 2013; Nawaz et al., 2014; Becherair, 2014; Santos, 2015) considered the role of institutions in economic performance of nations besides Solow's variables.

Most of the empirical works documented a significant cause-effect relation between intuitions and economic growth of countries (e.g. Vijayaraghavan and Ward, 2001; Becherair, 2014; Nawaz et al., 2014). Some others (e.g. Angeles, 2010; Santos, 2015) conclude lack of such causal relationship. The mixed findings created a puzzle in institutions-growth literature. Angeles' (2010) work differs from other studies, among others, by excluding control variables from the model. Angeles argued that the consequence of excluding the control variables, possibly omitted variables bias whose effect is either zero or positive, will not pose a problem as long as institutions indirectly influence these variables. However, this claim was not empirically tested. Santos (2015) studied effect of financial development on economic growth in SSA countries and also reports lack of casual link. Further, in some growth-institutions nexus studies, SSA countries were excluded from country set (e.g. Angeles, 2010). The underlying reasons include that these countries are trapped in poverty and institutions may not have a meaningful role on growth regardless of their quality level. On the other side, despite the resounding focus of researchers on institutions-growth nexus, few studies (e.g. Valeriani and Peluso, 2011; Sarwar et al., 2013) empirically investigated how institutions impacted growth at different stages of development.

The earlier works indicate usage of multiple estimation techniques and data sets in the study of the nexus between economic growth and institutions. In some of the studies (e.g. Vijayaraghavan and Ward, 2001; Sarwar et al., 2013; Becherair, 2014) the estimation techniques used do not address the common econometric problems of mutual causation, heterogeneity, and endogeneity. In studies employing the most reliable method available, variants of GMM (e.g. Angeles, 2010; Santos, 2015), current work contends concerns in regards to data set and selection of variables. Governance and development indicators from world bank used in Santos (2015) are evaluated as not best in reflecting North's notion of institutions (Becherair, 2014). In addition Santos (2015) used data set that does not fulfill fewer time period relative to number of cross sections requirement in the generalized method of moments (GMM) estimation technique developed by Arellano and Bond (1991), Arellano and Bover (1995) and Blundell and Bond (1998).

To address the gaps indicated and advance the debate in the field, this paper emphasizes the identification and use of appropriate estimation technique and data set in a study to determine the cause-effect link between institutions and economic growth of SSA countries. The research considered a more homogenous country set and institution variables with relatively less subjectivity bias and one that captures executive functions, which normally signal the performance of other dimensions of intuitions quality. In this regard the Polity IV Project based data were generated through a process with at most effort to measure North's notion of institutions, limits on executive power (Becherair, 2014) and noted to be most reliable of the lot (Glaeser et al., 2004). Fitting multiple models for control and institution variables, study provides information on individual and combined effect of institutions and control variables in explaining economic growth differences. In doing that, it empirically tests Angeles' thesis - whether control variables determine the relation between economic growth and institutions. The structure of the remainder of the paper follows: Section 2 gives insights from literature; Section 3 presents data and estimation technique; Section 4 details results and discussion, and Section 5 provides conclusion and recommendation.

## 2 Institutions and economic growth: literature review

With the ascent of Institutional Economics as a discipline, mere focus on market for efficiency gains through setting the ‘right’ price and thus obtaining optimal allocation of resources is waning. Rather, institutions, as institutional economists argue, are the fundamental drivers of differences in economic growth of countries. Availability, nature and functioning of institutions impact the organization of production through their effect on investments in human and physical capital. Hence, development is a process of organizational change; it is not the mere accumulation of capital (Sarwar et al., 2013).

North defined institutions as “the humanly devised constraints that shape human interaction” (North, 1990). In further explanation, he describes institutions as “the interaction between institutions and organizations that shapes the institutional evolution of an economy. As institutions are the rules of the game, North notes, organizations and their entrepreneurs are the players” (North, 1996, p. 345). In economists preference, institutions are disparate set of factors in the realm of informal (norms to values) and formal (e.g. property rights) institutions that together shape the behavior of organizations which affect economic performance (Williamson, 1975; Haggard, 2004; Becherair, 2014).

Henisz (2000) employed ordinary least squares (OLS), generalized least squares (GLS), and generalized method of moment (GMM) variant techniques to study the effect of institutions on economic growth. Using GDP growth, the study found executives-turnover yielded no significant effect on growth in OLS and GLS but a negative and significant effect in the GMM model. Index for factors from political constraint had a significant effect on growth in all methods used. Vijayaraghavan and Ward (2001) studied growth-institutions relation for country sets involving developed and developing economies. Result from the OLS method showed security of property rights and size of government significantly affect economic growth of nations.

Becherair (2014) used the variables including Governance, Security of property rights, Political freedom, and Government consumption. Separate OLS models fitted for panel data one without institution variables and the other model included institution and control factors together. Two of the institution factors, security of property rights (+) and government consumption (−), were found to be the most important factors to affect growth. The signs in the parenthesis indicate the direction of relationship. Further, parameter for oil dummy was found positive and significant. This validates the caution by Angeles (2010) that oil exporters may experience faster growth perhaps irrespective of institutions quality.

Similar to most literature Angeles (2010) used GDP growth rate in panel regression with fixed effect. Angeles excluded control factors from the model, which according to him the control factors themselves are the outcome of institutions. The control (commonly used: investment rates, human capital, trade openness, government expenditures, inflation etc.) were not included in the model and his argument is that including control factors would simply remove some of the effects to be captured by the institutions. In the dynamic GMM estimation procedure, Angeles’ consideration of longer time period allowed to account for inter-temporal changes by dividing dataset into sub-time periods maintaining sufficient number of observations per sub-samples. He also fitted the model by excluding fuel-dependent countries (suspected of faster growth regardless of institutions quality) and Sub-Saharan Africa and Latin America countries (suspected of being trapped in poverty hence institutions role obscured). In the end, Angeles (2010) finds that there is no evidence that countries with higher institutional quality experienced faster economic growth.

Sarwar et al. (2013) studied the importance of institutions in explaining growth differences among Asian economies. Authors followed various grouping or composition of institutions: formal institutions, informal institutions, financial institutions and legal institutions. Authors fitted fixed effect and random effect models and run different scenarios corresponding to the effect of economic variables, effect of economic variables and financial institutions, and effect of economic variables and all measures of institution identified. Financial institutions have significant and positive impact on growth while other variables are insignificant. In the scenario aimed at measuring the relative effect of all institution variables, the study found that all had significant and positive effect except the banking (from financial institutions) which related negatively to economic growth. However, common econometric problems of mutual causation, inter-temporal change and heterogeneity of included economies were not addressed in this study.

Roy et al. (2014) studied the role of institutions in determining growth for the relatively better developed states of India and made comparisons with one reference state. Authors identified components of institutions as (1) Legal Institution, (2) State intervention and (3) Political Institutions. With data obtained from various secondary sources compiled over seven yearly time periods, they developed indices using Principal Component Analysis (PCA) to address the problem of multicollinearity. The indices regressed on to the per capita income, annual state gross domestic product (GDP) growth and Extent of industrialization using the OLS estimation technique. Their findings indicated state intervention is significant in explaining the variations of growth of the states GDP, while both legal institutions and political institutions significantly affected the extent of industrialization across the states.

The review suggests the last two decades were the era where institutions-growth nexus investigations intensified. In the above review multiple estimation techniques were used to fit institutions-growth models. The studies used one or a combination of OLS, GLS, and GMM variants. Studies employing least squares based estimation techniques are prone to econometric problems. Regarding choice of type of response variable there were studies using GDP growth (e.g. Henisz, 2000), growth of GDP per worker (e.g. Becherair, 2014), and growth of GDP per capita (e.g. Angeles, 2010). Findings on institutions–growth nexus, though in agreement for the relatively earlier works that institutions matter for growth, a bold source of caution and thus puzzle surfaced by Angeles (2010) and Santos (2015) who found no causal relationship. It is also noted direction of causality between growth and institutions, and the channel of influence of institutions on economic development is not well understood and tools on how to address such issues are not providing consistent results across various works.

## 3 Methodology

This study examines institutions-economic growth nexus using 43 countries in SSA. Table A1 (in appendix section) provides the list of countries together with their economic development status (World Bank Group, 2019). A great deal of concern was already noted in growth-institutions studies. The literature was burdened with serious problems surrounding data, methodology, and identification (Aron, 2000). So, current work emphasized selection of method and data set in growth-institution research.

### 3.1 Data

The empirical study is based on a panel data set covering 43 SSA countries over the period 2002 to 2014. Study used secondary data obtained from world governance indicators WGI (2018), Maddison (2018), and Marshall and Jaggers (2018) databases. Two sets of explanatory variables were used: control variables; and the institution variables. Per capita real GDP growth was drawn from Maddison Project Database, version 2018 (Maddison, 2018). Attempt is made to identify institution quality indicators that measure how effectively existing rules are implemented. This is because indicators with mere description of political and economic institutions (e.g. presence/absence of constitutional rights) have nothing to say about performance of institutions (Aron, 2000). Therefore, study used the indicators that measure institutions quality (e.g. those related to respect for contracts, property rights) not those that merely describe the attributes and characteristics of political institutions (e.g. government type). Accordingly, Executive constraints (xconst), rule of law (ruloflaw) and Corruption Control (Corrupc) are the three institution variables extracted from two sources. Executive constraint indicates the extent of executive power constraints by political parties. It was obtained from Polity IV database (Marshall and Jaggers, 2018). The Polity IV offers a longer time period data set and the data generated are based on assessments by political scientists on diverse aspects of political institutions of countries. The variable, representing the constraints on the decision-making powers of chief executives, also better reflects North's notion of institutions. The executive constraints scores range from 1 to 7^1^ (Marshall and Jaggers, 2018).

Both Rule of Law and Control of Corruption were derived from world governance indicators database, 2018 update (WGI, 2018). Rule of law refers to perceptions on the extent of confidence in the rules in the society indicated by quality of contract enforcement, property rights, police and the courts, and the likelihood of crime and violence. Control of corruption is intended to capture the perceptions on the extent of power exercise for ‘private gain’ and state of elites to ‘capture’ the state for private interest (Docquier, 2014). Both, Rule of law and Corruption control variables, are measured with scores that ranges from −2.5 (weak) to 2.5 (strong) governance performance indicators. Three control variables namely share of government consumption (csh_g) at current PPPs, Exchange rate (xrate) in national currency/USD, and Human capital index (hc) were taken from Penn World Table version 9.0 (Feenstra et al., 2015). Human capital, from among Solow's growth variables, is preferred in current study given its especial importance^2^ in determining investment in and use of physical capital in developing countries.

### 3.2 Model specification and estimation technique

The empirical model for current study draws from Docquier (2014) shown in Eq. (1). The model is within the framework of neoclassical growth model (Solow, 1956) (from Mankiw et al., 1995) augmented to include measures of institutional quality.

(1) $${lny}_{it}=\alpha +{\beta \times Inst}_{it}+\sum_{k}\gamma^{k}{Control}_{it}^{k}+\varepsilon_{it}$$

where ${lny}_{it}$ is the log of GDP per capita in country i and year t, ${Inst}_{it}$ is a set of measures of institutional quality in country i and year t, ${Control}_{it}^{k}$ is a vector of k other explanatory variables, $\varepsilon_{it}$ is the error term, and ($\alpha ,\beta ,\gamma^{k}$) a vector of parameters to be estimated.

Use of appropriate estimation technique is important to obtain robust estimates for parameters capturing relations. Docquier (2014) notes the importance of being appropriate on dataset and size of nations included in cross-country studies. He argued that the inertia for institutions to change in quality is strong and the effect on development manifests slowly. Further, Docquier advises growth-institutions nexus studies use models with country-specific fixed and time effects especially in a data set having fewer number of control variables. From estimation technique viewpoint, this implies the need to include control variables in growth-institutions analysis.

Efendic and Pugh (2007) indicated pending concerns for further investigation surrounding analytical methods concerning mutual endogeneity (between institutions and economic growth), heterogeneity where cross-country studies are prone to, and proper understanding and measurement of transmission channels of cause-effect relations between institutions and development measures. Further caution relates to North's emphasis that formal institutions are the crystallization of informal institutions (North, 1990) where he urges the necessity of including informal institutions in the institutions-growth nexus studies.

For reliable and robust inferences out of institutions-growth analysis, current study uses a variant of GMM estimation techniques by Arellano and Bond (1991), Arellano and Bover (1995) and Blundell and Bond (1998). These estimators were developed to suit well panel data which fulfills the properties of (1) fewer time periods (T) and more cross sections (N) (N > T); (2) single left-hand-side dynamic variable which depends on its lagged values in a linear functional relationship; (3) independent variables are not strictly exogenous; (4) model with fixed individual effects; (5) study involving heteroskedastic data set; and (6) data set with autocorrelation within individuals but not across them (Arellano and Bond, 1991; Arellano and Bover, 1995). The GMM estimators also require fewer assumptions in data-generating process and suitable for dynamic and complex relations (Roodman, 2009; Arellano and Bond, 1991). The GMM estimators begin with general data-generating process given in Eq. (2).

(2) $$y_{it}={\alpha y}_{i\;t-1}+X_{it}^{\prime }+{\;\varepsilon }_{it}$$

$$\varepsilon_{it}=\mu_{i\;}+v_{it}$$

$$E\left[\;\mu_{i\;}\right]=E\left[v_{it}\right]=E\left[\mu_{i\;}v_{it}\right]=0$$

In the equation, disturbance term has two orthogonal components: the fixed effects, μ_i_ and the idiosyncratic shocks, $v_{it}$. The expression above can be written in different form given in Eq. (3) below.

(3) $${\Delta y}_{it}={\left(\alpha -1\right)y}_{i\;t-1}+X_{it}^{\prime }+{\;\varepsilon }_{it}$$

The alternative expression in Eq. (3) allows us to consider the growth concept of the response variable. The transformation in Eq. (3) through differencing removes the fixed effect component of the disturbance term. This procedure of transforming through differencing leads to a variant of GMM estimation technique known as Difference Generalized Method of Moments (DGMM). Nevertheless, the DGMM comes with two weaknesses: Endogenity because of lagged dependent variable included; and tendency to magnify the gaps in the unbalanced panels as a result of differencing procedure (Roodman, 2006).

To overcome the drawbacks of DGMM method, Arellano and Bover (1995) developed alternative method, “forward orthogonal deviations” or “orthogonal deviations” transformation. The procedure subtracts average value of future observations of the variable instead of subtracting the previous observation from the contemporaneous one. This gives us the augmented Generalized Method of Moments known as system GMM. Current study uses the system GMM estimation technique to test if institutions influenced economic growth for Sub-Saharan Africa countries. Guided by Hausman test result, the fixed effects model is preferred. Building on the works of Docquier (2014), the empirical model for the study is specified in Eq. (4) below.

(4) $${lnY}_{it}={\emptyset lnY}_{i\;t-1}+\beta I_{it}+\theta Z_{it}+\mu_{i}+T_{i}+{\;v}_{it}$$

$Y_{it}$ – per capita GDP, $Y_{i\;t-1}$ lagged per capita GDP, I – vector of institutional variables, Z vector of control variables, μ – unobserved country-specific fixed effects, T-time trend; ∅ , β and θ are parameters; *i-*number of cross sections (=1…N); *t –* number of time series (=1…T) and ${\;v}_{it}$ is the error term. In the above model specification^3^ lagged per capita GDP is endogenous variable and others are treated as weakly exogenous (*I*) and strictly exogenous (*Z*). The control variables are included to determine their combined effect on economic growth together with institutions. In addition, a model without these control variables is also estimated to test the thesis that the control variables are the ‘deep’ outcomes of institutional variables.

The GMM estimators from design meant for general use and consider instruments to address the endogeneity problem. The GMM instruments also known as ‘internal instruments’ are based on lags of the instrumented variables (Roodman, 2009). A Two-step system GMM is a variant of Orellando-Bond estimators and preferred in this study for it is more efficient and robust to heteroscedasticity and autocorrelation problems (Roodman, 2009). With the assumption of no autocorrelation across individuals in a panel, time dummies in the system GMM are considered to purge time-related shocks from the errors (Roodman, 2006). The two-step system GMM was fitted with STATA version 14.

System GMM reliability requirements underlie two specification tests. Hansen test for over-identifying restrictions through checking the overall instruments validity test (null: no over identification restriction). The second test is on serial correlation of error terms for first-and second-order autocorrelation (null: no serial autocorrelation exists) (Arellano and Bond, 1991).

## 4 Results and discussion

### 4.1 Descriptive statistics

Table 1 gives summary statistics of the variables in the model. Country specific figures are given in Table A2 in the appendix section. Average per capita GDP growth rate for the period 2002–2014 was 2.58% with strong variation among countries (Table 1). Chad experienced the fastest (8.12%) growth while Central African Republic had a declining period average (−3.24%) (Table A2). The executive constraint score (4.18) indicates, on average, SSA countries fall in the category where the countries already passed the status of only exerting a ‘Slight to Moderate Limitation on Executive Authority, score 3’ but not reached the level of exercising a ‘Substantial Limitations on Executive Authority, score 5’. Comoros averaged the highest score of 6.69 over the period indicating its citizens were able to exert control power over the executive in most areas of the activities. Equatorial Guinea had mean score executive constraints of 1 (one) which places the country among those with no regular limitations on the executive's actions. Period's average score for rule of law and corruption control were -0.7 and -0.65, respectively. Highest score for rule of law (0.96) recorded for Mauritius and the least (−1.6) for D.R. of the Congo. In regards to corruption control in SSA for the panel, the countries Angola (−1.3) and Botswana (0.97) appeared the weakest and strongest in the country set, respectively.

**Table 1:** Summary statistics of variables.

Variables	Obs.	Mean	Std. Dev.	Min	Max
Per capita GDP (gdpc)	559	4297.809	6785.542	520	48334
Per capita GDP growth (%)	559	2.577	5.031	-38.1	29.199
Executive constraint (xconst)	547	4.176	1.911	0	7
Rule of Law (ruloflaw)	559	-0.696	0.627	-1.850	1.080
Control of Corruption (Corrupc)	559	-0.651	0.602	-1.770	1.220
Institution quality Index (Instdx)	559	0	1	-1.860	3.109
Human capital index (hc)	481	1.737	0.413	1.088	2.809
Gov. consumption share (csh_g)	559	0.174	0.120	0.017	0.954
Exchange rate (xrate)	559	570.207	971.129	0.792	7014.119

Source: Author's Computations.

Rule of law and corruption control, both measured with the same scale, are obtained from same source. In order to determine the combined institutional quality impact on economic growth, institution quality index (Instdx) computed from rule of law and corruption control. Countries Botswana and Equatorial Guinea had the highest (2.67) and lowest (−1.5) index figures respectively.

A look at the correlation matrix (Table 2) provides an informative cursory on the relationship between the variables. All institution variables, together with index derived from them, revealed a mild but positive association with GDP per capita. Among control variables, human capital index had a positive and relatively strong correlation with per capita GDP. Share of Government consumption and exchange rate had a negative and weaker association with GDP per capita. The figures in Table 2 also show that exchange rate had a negative association with both economic and institutional quality indicators. The negative association with per capita GDP indicates devaluation in the value of local currency could have a contractionary effect.^4^ In relation to institutions, poor quality status (e.g. political instability possibly due to rampant corruption) devalues country's currency exchange. For instance, when there is political instability, there will be less foreign investments, which leads to limited supply of foreign currency. Correlation coefficient among institutional variables, which ranged between 49.2 to 87.9% (Table 2), is indicative of the appropriateness of deriving a composite variable, the institution index, to study the combined impact of institutions on growth.

**Table 2:** Correlation matrix.

	gdpc	xconst	ruloflaw	Corrupc	Instdx	hc	csh_g	xrate
Per capita GDP (gdpc)	1							
Executive constraint (xconst)	0.202	1						
Rule of Law (ruloflaw)	0.482	0.557	1					
Corruption control (Corrupc)	0.426	0.492	0.879	1				
Institution Index (Instdx)	0.426	0.492	0.879	1	1			
Human capital index (hc)	0.664	0.358	0.401	0.382	0.382	1		
Gov. consumption share (csh_g)	−0.025	0.169	−0.055	0.014	0.014	0.002	1	
Exchange rate (xrate)	−0.243	−0.004	−0.136	−0.205	−0.205	−0.222	0.185	1

Source: Author's Computation.

### 4.2 Econometric results

Table 3 shows the results of data diagnosis to check for econometric problems in a pooled OLS model. Overall model fit is good with explanatory power of 99.78 as indicated by the adjusted R-square. Besides the strongly significant lagged dependent variable, three of the explanatory variables (one from institution and two from control variables) significantly affected per capita GDP at 5% significance level. The pooled OLS model^5^ did not pass the basic specification tests (Table 3). Supporting the use of system GMM, tests for heteroscedasticity and omitted variables rejected the lack of specification problems of the OLS method. The third test is whether the model suffers from serial autocorrelation problem. Test statistics reported in Table 3 indicate the presence of first and second order serial autocorrelation. Hence, a two-step system GMM is appropriate to remedy the problem. The Hauseman test^6^ result also confirms fixed effect model is appropriate for the data set. The next paragraphs present system GMM estimation results.

**Table 3:** Pooled OLS Model tests.

Econometric Problems	Test and Null	Statistics
Heteroscedasticity	Breoush-Pagan/Cook-Weisberg	Chi2 = 24.52
	H_o_: Constant variance	Prob > chi2 = 0.0000
Omitted variable	RESET	F (3, 411) = 2.49
	H_o_: no omitted variable	Prob > F = 0.0595
Autocorrelation, AR (1)	Arellano-Bond	Z = 5.24
	H_o_: no first order serial autocorrelation	Prob > z = 0.0000
Autocorrelation, AR (2)	Arellano-Bond	Z = 4.17
	H_o_: no second order serial autocorrelation	Prob > z = 0.0000

Source: Author's Computation.

Table 4 presents a summary of the dynamic models results. Column [1] is the result from system GMM model^7^ that contains all the institution and control variables specified. The independent effect of each of the institution variables is given in columns [2] to [4]. The fifth column [5] gives the combined effect of institution quality through the index computed from individual variables. In addition, the sixth column [6] gives system GMM result with control variables excluded. In all the columns, the log of the lagged per capita GDP is statistically significant at 1%. The finding evidences the persistency of per capita GDP growth in SSA region.

**Table 4:** System GMM Estimators (Dependent Variable: per capita GDP (log)).

VARIABLES	[1]	[2]	[3]	Models [4]	[5]	[6]	[7]
Constant	1.341 (2.822)	0.0806 (1.034)	2.722 (2.371)	2.416 (2.155)	2.272 (2.222)	−2.567 (5.029)	−12.076 (11.132)
Lagged GDP per capita (log)	0.988∗∗∗ (0.167)	1.015∗∗∗ (0.053)	0.797∗∗∗ (0.291)	0.710∗∗∗ (0.250)	0.710∗∗∗ (0.250)	0.984∗∗∗ (0.052)	0.837∗∗∗ (0.220)
Executive constraint (xconst)	−0.0308 (0.084)	0.015 (0.031)				0.0186 (0.025)	
Rule of Law	0.0744 (0.303)		0.0694 (0.095)			−0.0534 (0.114)	
Control of Corruption	−0.0594 (0.313)			0.221 (0.228)		0.196 (0.175)	
Institution quality Index					0.133 (0.137)		0.180 (0.125)
Government consumption Share	0.0325 (0.410)	0.079 (0.173)	0.137 (0.480)	0.18 (0.567)	0.180 (0.576)		
Exchange rate	−0.0617 (0.089)	−0.0255 (0.036)	−0.0969 (0.100)	−0.0843 (0.114)	−0.084 (0.114)		
Human capital index	−0.434 (0.719)	−0.0678 (0.220)	−0.347 (0.573)	0.175 (0.949)	0.175 (0.949)		
Observations.	433	433	444	444	444	505	516
Groups	37	37	37	37	37	43	43
Time dummies	yes	yes	yes	yes	yes	yes	yes
Instruments	24	24	24	24	24	24	24
F-statistic	141.27	780.01	89.98	37.16	37.16	270.21	30.52
GMM instrument Lag	1	1	1	1	1	1	1
AR (1)	0.122	0.056	0.175	0.12	0.120	0.017	0.041
AR (2)	0.849	0.437	0.814	0.557	0.557	0.129	0.161
Hansen test	0.503	0.203	0.67	0.634	0.634	0.417	0.819

*Note*: Robust options used; Corrected Std. Err*.*; ∗∗∗p < 0.01, ∗∗p < 0.05, ∗p < 0.1 indicate significance at 1%, 5% and 10% respectively.

*Source*: Author's computations.

Estimates in columns [2] to [4] capture the individual effect of institution variables on economic growth. Accordingly, all institution variables had no statistically significant effect on economic growth. In the order of control of corruption, rule of law and executive constraints, the variables effect on growth diminished. In column [5], an attempt to test the combined effect of the institutional variables proved the same: index of institutions variable had no significant effect on growth. Model outputs in column [6] are intended to examine whether the control variables affect roles of institutions in growth-institutions relations as implied by Angeles (2010). All the three variables relation with growth are statistically insignificant as when the control variables included, column [1]. Individual coefficients in column [6] indicate the effect of executive constraint and control of corruption improved without control variables. On the other hand, Rule of law did not only get weaker in role but also had a negative association with growth. Apart from possible bias from measurement owing to subjective assessment of the variables, different reasons can play to the incidence. Measured with same scale and coming from same source, the period mean score for the rule of law was 6.5% behind the score of control of corruption (Table 1). Perhaps the rule of law would have a higher value (score) in the institutional threshold levels for its effect on growth to set in. In general, except for rule of law, column [6] results indicate improvement in the role of institution to determine growth in the absence of control variables. This was further strengthened by column [7] results where the coefficient for index of institution variables improved under no control variables scenario. Thus, this finding provides empirical proof that control variables are possibly the ‘deep’ outcomes of institutions and should not be considered as direct factors to explain economic growth of nations.

In regards to model validity assessment, Table 4 also presents key statistics on the test reports. In all the scenarios considered, the models performed well concerning overall significance, at 1% indicated by F-statistic and specified correctly given the number of instruments stayed below the number of individuals. Both tests validated the model. The p-values for over-identifying restrictions tests failed to reject each null hypothesis that instruments are exogenous at 10% significance level. The second order serial autocorrelation tests also maintained the null hypothesis of no serial autocorrelation in all the cases at 10%. The first order serial autocorrelation tests failed to reject the null hypothesis except in three cases, one at 10%, and the two cases at 5%. When the autocorrelation tests fail to reject first order autocorrelation, it is expected to have such result in dynamic model where lagged dependent variable is included as one of the explanatory variables. In general, with the preferred lag length of one year, the specification test result for AR (2) indicated no second-order serial autocorrelation. Hansen test result also showed no over-identification of the instruments used in the models. Hence, the models fitted lend the study result reliable for making inferences.

#### 4.2.1 Discussion

Institutions–growth studies appear intensified over the past two decades. Yet, an emerging development in the literature is the divergence of conclusions on whether there is casual link between institutions and economic growth. While earlier works evidenced institutions matter for economic growth of nations, fewer and relatively recent studies find institutions have no significant impact on growth. The current work, which studied economic growth-institutions nexus in SSA countries, finds institutions make no significant impact on growth. This is in contrary to earlier findings that there is a casual relationship (e.g. Vijayaraghavan and Ward, 2001; Kilishi et al., 2013; Becherair, 2014; Nawaz et al., 2014). However, current finding has backing from literature. Santos (2015) and Angeles (2010) concluded no statistically significant causal relationship. It confirms the unsettled state of literature on whether institutions matter for growth. Indicative evidence that the impact of institutions on growth improved without control variables is the second key finding of current work. This gives empirical backing to Angeles' (2010) position that the control variables are the outcome of institutions and should not be considered as direct causes of economic growth. The next paragraphs discuss literature-based reservations in institutions-growth nexus studies and forwards future research focus areas.

The institutions-growth literature, after great stride to identify and measure institution's role for economic growth of nations, there is still muddy aspects regarding whether institutions really matter for economic growth of nations. Theoretical explanations and empirical works indicate pending reservations underlying the need for further scrutiny of growth-institutions links. The reservations emanate from one or combination of (1) choice of variables (data set), (2) inclusion of informal institutions and (3) considering threshold for institutions quality indicators. The last two were noted most important for developing country conditions. In cross-country regressions to study growth differences, there are admissible limitations associated with variables considered. In this regard Mankiw et al. (1995) notes the result of studies is contingent on the choice of variables. Further, several data sources with corresponding variables are used. Definition and measurement of institutions, especially political and economic institutions, is not clear for they are interwoven and can either be inclusive or extractive (Acemoglu and Robinson, 2013). Surveys and subjective assessments towards the construction and measurement of these variables could cause some form of bias. This happens for various reasons. Most importantly, the constitutional measures may be noisy, ‘rules on the book’ of the countries and practices on the ground can be quite different, and measures of constraints used by scholars may not, at least strongly, reflect constitutional constraints raising doubts on effectiveness of changing political rules (Becherair, 2014).

Particular to developing nations, the informal institutions need to be included in growth – institution nexus studies. The literature, however, indicates a disproportionate focus is given to formal institutions. The informal institutions such as culture, religion, historical events and legal origins, play a role for extended time period and they determine efficiency of formal institutions (Vītola and Senfelde, 2012). Hence, not accounting for informal institutions, pervasive in the developing countries, could have underrepresented institutions roles in growth determination. The third reservation hinges from contemporary development in growth-instructions literature. Empirical evidence suggests possible presence of thresholds on institutions quality measures that signals the start (possibly the phase-out) of institutions quality levels to affect economic growth. Law et al.’s (2013) study on ‘Institutional quality thresholds and the finance – Growth nexus’ reported that financial development-growth nexus was dependent on institutions level of development. This development implies that the relations can only be realized when quality of institutions exceeds a certain threshold level. In SSA countries, where institutional frameworks are weak, it is quite possible that institution measures are lower and well below the thresholds. Such possibilities are noted in the literature where selection of countries to study institutions-growth link in cross-region studies considers the income level. In Angeles (2010), for instance, SSA countries were excluded from studied country set for the reason these countries are trapped in poverty and institutions may not have a meaningful role for their growth.

Current work emphasized selection of indicators, data set and estimation technique of models to study institution-growth link in SSA countries. Yet, lack of indicators from informal institutions, pervasive in study region, constitutes the main limitation of the research. North emphasized the role of informal institutions as direct factor to growth or in affecting the formal institutions. Difficulty in measurement and thus lack of information on indicators for informal institutions hindered their inclusion in the model. Hence, future research, among others, need to work on integrating indicators or proxies for informal institutions in order to better explain the reasons for economic growth disparity of nations. Further work is also needed in areas (1) to clearly identify indicators that measure institutions quality as reflected in performance metrics; and (2) to ascertain the presence of thresholds on measures of these indicators and incorporation in the growth-institution models.

## 5 Conclusion and recommendation

The literature underlines several variables, data sets and estimation methods used to study institutions-growth nexus. Most important aspect and noteworthy is that the literature is unsettled when it come to the conclusions. There is divergence of conclusions on whether institutions really influence economic growth. In this paper, institutions-economic growth nexus studied using a panel of 43 SSA countries over a period of thirteen years, 2002 to 2014. The study included institution measures from most reliable data set and employed a system GMM estimation technique that is noted to address econometric problems in cross-country regressions. The findings illustrate the persistency of per capita GDP growth in the region as indicated by the positive and significant coefficient of the lagged per capita GDP. The results from a version of system GMM model reveal indicative evidence that excluding control variables in the model improves the role of institution variables. This provides empirical backing to the claims that economic factors are the outcomes of institutions development. It is also showed that institutions had no significant impact, whether in combination through their index or individually, on economic growth of countries in the region. This finding lends support for the independent hypothesis postulates that institutions and economic growth are casually independent. The research also identifies further work in two areas in relation to reservations still discussed in the literature. One is on the measurement and thus inclusion of the informal component of institutions. The second is to substantiate the presence and thus identification of thresholds for institutions quality.

## Declarations

### Author contribution statement

Milkessa Temesgen Chomen, PhD candidate: Conceived and designed the experiments; Performed the experiments; Analyzed and interpreted the data; Contributed reagents, materials, analysis tools or data; Wrote the paper.

### Funding statement

This work was supported by 10.13039/501100016392Ambo University.

### Data availability statement

Data associated with this study has been deposited at The Worldwide Governance Indicators, WGI. 2018. “The Worldwide Governance Indicators, 2018 Update: Aggregate Governance Indicators 1996–2017.” www.govindicators.org.

Maddison, A. 2018. “Statistics on World Population, GDP and per Capita GDP.” Maddison Project Database, version 2018. www.ggdc.net/maddison. Marshall, M. G., and K. Jaggers. 2018. “Polity IV Project: Dataset Users’ Manual.” www.systemicpeace.org.

### Declaration of interest's statement

The authors declare no conflict of interest.

### Additional information

No additional information is available for this paper.

## Footnotes

1 A value of 1, for instance, corresponds to the case of “Unlimited Authority” and would be assigned under conditions such as “There is no legislative assembly, or there is one but it is called and dismissed at the executive pleasure”. A value of 7 corresponds to the case of “Executive Parity or Subordination” and its overall description is “Accountability groups have executive authority equal to or greater than the executive in most areas of activity”. Values 2, 4 and 6 are just described as “intermediate category” (Marshall and Jaggers, 2018).

2 “Having little human capital, poor countries invest little in tangible capital, If virtually all income is attributable to this capital, human and nonhuman, a dearth of human capital has a devastating effect on national potential output “(Mankiw et al., 1995).

3 Instrumental variables used: GMM instrument is one-period lagged values of the per capita GDP.

4 Krugman and Taylor's (1978) condition for contractionary effect of devaluation, initially imports exceeding exports, aligns with developing countries situation and confirms possible negative association between exchange rate and growth.

5 STATA output for the pooled OLS model given in the appendix section, Table A4.

6 STATA output for the test is included in the in appendix section, Table A5.

7 STATA output with details included in appendix section, Table A3.
