# Supplementary material for: Institutions – Economic Growth Nexus in Sub-Saharan Africa
Source: Heliyon. 2022 Dec 15;8(12):e12251. doi: 10.1016/j.heliyon.2022.e12251 (PMC9800297; doi:10.1016/j.heliyon.2022.e12251)
Supplement: Appendix_supplementary file [file mmc1.docx]

# Appendix

Table A1: Sampled Countries by Economic Development class^a^

| LOW-INCOME  ($995 OR LESS) | | LOWER-MIDDLE  ($996 - $3,895) | UPPER-MIDDLE  ($3,896 - $12,055) |
| --- | --- | --- | --- |
|  | |  |  |
| 1. Benin 2. Burkina Faso 3. Burundi 4. Central African Republic 5. Chad 6. Comoros 7. D.R. of the Congo 8. Ethiopia 9. Gambia 10. Guinea 11. Guinea-Bissau 12. Liberia 13. Madagascar | 1. Malawi 2. Mali 3. Mozambique 4. Niger 5. Rwanda 6. Senegal 7. Sierra Leone 8. Tanzania 9. Togo 10. Uganda 11. Zimbabwe | 1. Angola 2. Cameroon 3. Cape Verde 4. Congo 5. Côte d'Ivoire 6. Ghana 7. Kenya 8. Lesotho 9. Mauritania 10. Nigeria 11. Sudan 12. Swaziland 13. Zambia | 1. Botswana 2. Equatorial Guinea 3. Gabon 4. Mauritius 5. Namibia 6. South Africa |
| **Total number of countries** | | | **43** |

^a^ classification of countries is based on the 2017 per capita income.

Source: World Bank Group (2019)

Table A2: Thirteen (13) Year Averages of Indicators

| **country** | **Growthpc** | **gdpc** | **xconst** | **ruloflaw** | **Corrupc** | **csh_g** | **hc** | **xrate** |
| --- | --- | --- | --- | --- | --- | --- | --- | --- |
| Angola | 6.923 | 6790.462 | 3.000 | -1.349 | -1.307 | 0.152 | 1.389 | 82.790 |
| Benin | 1.068 | 1841.923 | 5.000 | -0.528 | -0.632 | 0.166 | 1.547 | 517.094 |
| Botswana | 3.228 | 12547.460 | 7.000 | 0.651 | 0.972 | 0.205 | 2.673 | 6.590 |
| Burkina Faso | 2.450 | 1244.231 | 3.000 | -0.441 | -0.287 | 0.212 | 1.149 | 517.094 |
| Burundi | 0.581 | 704.308 | 6.000 | -1.154 | -1.115 | 0.237 | 1.282 | 1212.183 |
| Cameroon | 0.973 | 2491.538 | 2.000 | -1.107 | -1.108 | 0.100 | 1.835 | 517.094 |
| Cape Verde | 2.528 | 5770.231 | 7.000 | 0.497 | 0.772 | 0.182 | . | 86.926 |
| Central African Republic | -3.244 | 839.615 | 2.538 | -1.451 | -1.118 | 0.123 | 1.456 | 517.094 |
| Chad | 8.128 | 1513.923 | 2.000 | -1.422 | -1.365 | 0.180 | . | 517.094 |
| Comoros | 1.293 | 1475.308 | 6.692 | -0.989 | -0.792 | 0.265 | . | 387.820 |
| Congo | 1.312 | 5678.692 | 2.000 | -1.215 | -1.108 | 0.126 | 2.017 | 517.094 |
| Côte d'Ivoire | 0.526 | 2650.000 | 2.000 | -1.242 | -0.989 | 0.118 | 1.515 | 517.094 |
| D.R. of the Cong | 3.345 | 633.462 | 4.500 | -1.612 | -1.375 | 0.531 | 1.610 | 659.212 |
| Equatorial Guinea | 5.354 | 39965.390 | 1.000 | -1.361 | -1.543 | 0.046 | . | 517.094 |
| Ethiopia | 5.633 | 1008.538 | 3.000 | -0.750 | -0.635 | 0.120 | 1.270 | 12.354 |
| Gabon | 0.285 | 16386.610 | 2.923 | -0.514 | -0.820 | 0.195 | 2.403 | 517.094 |
| Gambia | 0.668 | 1668.154 | 2.000 | -0.432 | -0.611 | 0.111 | 1.428 | 28.929 |
| Ghana | 4.480 | 2835.154 | 6.000 | 0.002 | -0.131 | 0.153 | 2.239 | 1.347 |
| Guinea | 0.923 | 1325.000 | 3.154 | -1.365 | -1.048 | 0.129 | . | 4760.788 |
| Guinea-Bissau | 0.494 | 1298.154 | 5.000 | -1.315 | -1.205 | 0.230 | . | 517.094 |
| Kenya | 1.772 | 2554.615 | 6.538 | -0.851 | -0.975 | 0.119 | 2.116 | 78.614 |
| Lesotho | 4.028 | 2066.538 | 7.000 | -0.168 | 0.055 | 0.278 | 2.080 | 8.060 |
| Liberia | 3.511 | 719.231 | 5.600 | -1.131 | -0.838 | 0.096 | 1.697 | 1.000 |
| Madagascar | -0.342 | 1366.615 | 5.077 | -0.574 | -0.376 | 0.462 | 1.620 | 1929.932 |
| Malawi | 2.119 | 997.154 | 5.769 | -0.186 | -0.602 | 0.111 | 1.688 | 177.270 |
| Mali | 1.786 | 1398.538 | 4.615 | -0.365 | -0.634 | 0.208 | 1.208 | 517.094 |
| Mauritania | 2.900 | 2958.616 | 2.615 | -0.807 | -0.597 | 0.295 | 1.623 | 272.524 |
| Mauritius | 3.082 | 14960.540 | 7.000 | 0.960 | 0.402 | 0.176 | 2.438 | 29.935 |
| Mozambique | 5.123 | 813.154 | 4.154 | -0.636 | -0.545 | 0.140 | 1.172 | 26.848 |
| Namibia | 3.386 | 7580.846 | 5.000 | 0.176 | 0.292 | 0.202 | 2.114 | 8.060 |
| Niger | 1.965 | 793.385 | 4.846 | -0.591 | -0.741 | 0.169 | 1.158 | 517.094 |
| Nigeria | 5.222 | 4440.769 | 5.000 | -1.198 | -1.174 | 0.083 | 1.695 | 139.559 |
| Rwanda | 5.296 | 1249.462 | 2.923 | -0.511 | 0.053 | 0.152 | 1.548 | 576.024 |
| Senegal | 1.239 | 2078.461 | 5.385 | -0.197 | -0.268 | 0.130 | 1.427 | 517.094 |
| Sierra Leone | 6.264 | 1427.615 | 5.615 | -0.988 | -0.920 | 0.144 | 1.477 | 3375.389 |
| South Africa | 2.006 | 11173.390 | 7.000 | 0.135 | 0.212 | 0.169 | 2.418 | 8.060 |
| Sudan | 2.855 | 3293.077 | 1.769 | -1.373 | -1.289 | 0.107 | 1.501 | 2.914 |
| Swaziland | 2.057 | 7067.153 | 2.000 | -0.628 | -0.341 | 0.154 | 1.667 | 8.060 |
| Tanzania | 3.497 | 2017.231 | 3.000 | -0.405 | -0.576 | 0.153 | 1.579 | 1311.975 |
| Togo | 0.639 | 1220.461 | 2.385 | -0.925 | -0.959 | 0.159 | 1.757 | 517.094 |
| Uganda | 3.454 | 1637.615 | 3.000 | -0.431 | -0.894 | 0.116 | 1.913 | 2080.743 |
| Zambia | 4.222 | 2783.231 | 5.000 | -0.423 | -0.474 | 0.108 | 2.227 | 4.702 |
| Zimbabwe | -2.207 | 1539.923 | 2.769 | -1.717 | -1.348 | 0.177 | 2.335 | 1.000 |
| Total | 2.577 | 4297.809 | 4.176 | -0.696 | -0.651 | 0.174 | 1.737 | 570.207 |

Growthpc: Per capita GDP growth (%); gdp: GDP per capita; xconst : Executive constraint; ruloflaw :Rule of Law; Corrupc: Control of Corruption; Instdx: Institution quality Index; hc : Human capital index; csh_g : Gov. Consumption share; xrate: Exchange rate

Table A3: Two-step System GMM (Dependent: Log of per capita GDP)

Table A4: Pooled OLS Result

Table A5: Hausman test

Table A6: Autocorrelation test, robust option
